# Is There Any Relationship between TSH Levels and Prognosis in Acute Coronary Syndrome?

**DOI:** 10.5935/abc.20180019

**Published:** 2018-02

**Authors:** Alexandre de Matos Soeiro, Victor Arrais Araújo, Júlia Pitombo Vella, Aline Siqueira Bossa, Bruno Biselli, Tatiana de Carvalho Andreucci Torres Leal, Maria Carolina Feres de Almeida Soeiro, Carlos V. Serrano Jr., Christian Mueller, Mucio Tavares de Oliveira Junior

**Affiliations:** 1 Unidade Clínica de Emergência - InCor - HCFMUSP, São Paulo, SP; 2 Universidade de Basel, Basel, Suíça

**Keywords:** Acute Coronary Syndrome, Thyrotropin/metabolism, Euthyroid Sick Syndromes, Hospital Mortality

## Abstract

**Background:**

Some small studies have related higher levels of thyrotropin (TSH) to potentially worse prognosis in acute coronary syndromes. However, this relationship remains uncertain.

**Objective:**

To analyze the outcomes of patients with acute coronary syndromes in relation to the value of TSH at admission.

**Methods:**

Observational and retrospective study with 505 patients (446 in group I [TSH ≤ 4 mIU/L] and 59 in group II [TSH > 4 mIU/L]) with acute coronary syndromes between May 2010 and May 2014. We obtained data about comorbidities and the medications used at the hospital. The primary endpoint was in-hospital all-cause death. The secondary endpoint included combined events (death, non-fatal unstable angina or myocardial infarction, cardiogenic shock, bleeding and stroke). Comparisons between groups were made by one-way ANOVA and chi-square test. Multivariate analysis was determined by logistic regression. Analyses were considered significant when p < 0.05.

**Results:**

Significant differences between groups I and II were observed regarding the use of enoxaparin (75.2% vs. 57.63%, p = 0.02) and statins (84.08% vs. 71.19%, p < 0.0001), previous stroke (5.83% vs. 15.25%, p = 0.007), combined events (14.80% vs. 27.12%, OR = 3.05, p = 0.004), cardiogenic shock (4.77% vs. 6.05%, OR = 4.77, p = 0.02) and bleeding (12.09% vs. 15.25%, OR = 3.36, p = 0.012).

**Conclusions:**

In patients with acute coronary syndromes and TSH > 4 mIU/L at admission, worse prognosis was observed, with higher incidences of in-hospital combined events, cardiogenic shock and bleeding.

## Introduction

Patients with severe nonthyroidal illness often experience concomitant disorders in thyroid function. In severe illness of nonthyroidal origin, including acute myocardial infarction (AMI), the thyroid hormone system may be down-regulated. These conditions can induce changes in one or more aspects of thyroid hormone economy, leading to findings referred to as sick euthyroid syndrome, which poses a diagnostic and therapeutic challenge for the clinician. The cardiovascular system is very sensitive to thyroid hormones, and a wide spectrum of cardiac changes has long been recognized in overt thyroid dysfunction.^1-3^

The real value of thyrotropin (TSH) as marker of prognosis in acute coronary syndromes (ACS) is still uncertain. Therefore, the objective of this study was to analyze the outcomes of patients with ACS related with the TSH value measured in the emergency department.

## Methods

### Study population

This was an observational, retrospective databank analysis study performed in a tertiary health centre with 505 patients with ACS included between May 2010 and May 2014. They were divided in two groups: TSH ≤ 4 mIU/L (group I, n = 446) and TSH > 4 mIU/L (group II, n = 59). Patients with known thyroid disorders were excluded.

All patients were diagnosed and treated according to the AHA/ESC Task Force guidelines.^4,5^ All patients underwent percutaneous coronary intervention less than 24 hours after onset of ACS.

The primary outcome was in-hospital all-cause mortality. The secondary outcome was major adverse cardiac events (MACE) including death (of any cause), non-fatal unstable angina or AMI/target vessel revascularization, cardiogenic shock, bleeding (major and minor), and stroke.

The study was approved by the ethics and research committee.

### Analytical methods

The following data were obtained: age, sex, diabetes, systemic arterial hypertension, smoking habit, dyslipidemia, family history of premature coronary artery disease, heart failure, previous coronary artery disease, previous stroke, hematocrit, creatinine, higher troponin, systolic blood pressure, left ventricular ejection fraction and medications used (within the first 24 hours) (Table 1).

Blood was sampled immediately after admission, prior to administration of medications (baseline) and daily, according to institution protocol. TSH was obtained routinely in all patients with ACS. Cardiac markers such as troponin-I were measured using standard clinical chemistry. Laboratory upper limits of normal were 0.04 ng/mL (99^th^ percentile) for troponin-I measured by *Elecsys 2010* (*Siemens Healthcare Diagnostics Inc., USA)* 4^th^ generation immunoassay.

Major bleeding was defined using BARC^6^ score types 3 and 5, and minor bleedings, types 1 and 2. Post-operative bleeding events were not considered.

### Statistical analysis

Descriptive analyses of data collected included median, minimum and maximum values. Categorical variables were described as percentages. Comparisons between groups were made by ANOVA one-way and chi-square test (to categorical variables), and a *p* value < 0.05 was considered significant. If Kolmogorov-Smirnov tests confirmed a normal distribution, continuous variables were presented as mean ± standard deviation, and were compared using Student *t* test for independent samples. Mann-Whitney *U* test was used to compare not normally distributed continuous variables, which were presented as median and interquartile range.

Multivariate analysis was determined by logistic regression, and a *p* value < 0.05 was considered significant. The patients' baseline characteristics are shown in Table 1.

All statistical procedures were performed using the statistical software SPSS, version 10.0.

## Results

**Figure 1:**
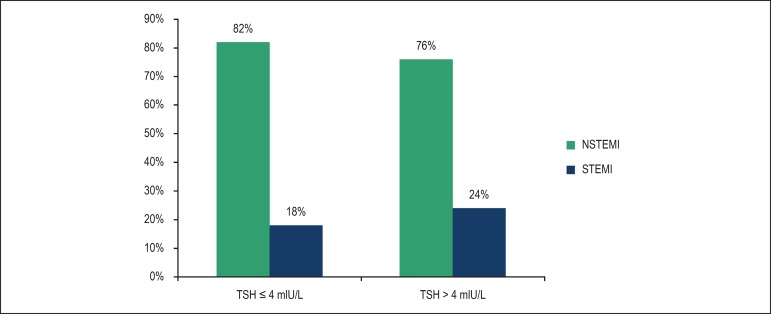
Classification of ACS according to TSH levels. NSTEMI: Non-ST-elevation myocardial infarction; STEMI: ST-elevation myocardial infarction; TSH: thyrotropin.

The median age was 63 years, and approximately 59% of patients were male. Baseline characteristics and univariate analysis are shown in Table 1. ST-elevation myocardial infarction (STEMI) was observed in 18% of group I versus 24% of group II (p = 0.08) (Figure 1).

**Table 1:** Baseline characteristics of patients according with TSH levels.

	TSH ≤ 4 mIU/L	TSH > 4 mIU/L	p
Age (mean)	62.5	66.3	0.86*
Male (%)	61%	51%	0.14^#^
Diabetes Mellitus (%)	39%	48%	0.38^#^
Hypertension (%)	80%	76%	0.49^#^
Smoking habit (%)	40%	37%	0.72^#^
FH of CAD (%)	13%	10%	0.56^#^
Dyslipidemia (%)	47%	48%	0.9^#^
Heart failure (%)	8%	10%	0.62^#^
Previous stroke (%)	6%	15%	0.007^#^
Previous AMI (%)	38%	48%	0.14^#^
Previous CABG (%)	18%	27%	0.08^#^
Previous PCI (%)	25%	32%	0.21^#^
Ht (%) (mean)	42.2	41.5	0.08*
Cr (mg/dL) (mean)	2.18	2.99	0.51*
SBP (mm Hg) (median)	134.5	133.8	0.24^π^
EF (%) (median)	42.5	33.7	0.62^π^
Troponin (higher) (ng/dL) (mean)	4.68	7.37	0.52*
ASA (%)	99%	93%	0.12^#^
Beta-blocker (%)	68%	54%	0.12^#^
Enoxaparin (%)	72%	58%	0.021^#^
ACE inhibitor (%)	51%	48%	0.64^#^
Statin (%)	83%	71%	< 0.001^#^

TSH: thyrotropin; FH: family history; CAD: coronary artery disease; AMI: acute myocardial infarction; CABG: coronary artery bypass grafting; PCI: percutaneous coronary intervention; SBP: systolic blood pressure; Ht: hematocrit; Cr: creatinine; EF: ejection fraction; ASA: acetylsalicylic acid; ACE: angiotensin-converting-enzyme;

# Q-square test;

* Student t test for independent samples;

π Mann-Whitney U test.

Multivariate analysis is shown in Table 2 and describes the differences between groups I and II in combined events (14.80% vs. 27.12%, respectively, OR = 3.05, p = 0.004), cardiogenic shock (4.77% vs. 6.05%, respectively, OR = 4.77, p = 0.02) and bleeding (12.09% vs. 15.25%, respectively, OR = 3.36, p = 0.012).

**Table 2:** Results of multivariate analysis of in-hospital outcomes comparing groups I and II

	TSH ≤ 4 mIU/L	TSH > 4 mIU/L	OR	95% CI	p
Reinfarction	1.3%	0%	0.2	0.11 - 3.45	0.37
Cardiogenic Shock	6.1%	13.6%	1.72	1.25 - 4.68	0.029
Bleeding	6.5%	15.3%	3.36	1.31 - 8.65	0.012
Stroke	0.9%	0%	0.9	0.15 - 9.32	0.9
Mortality	3.1%	8.5%	2.32	0.63 - 8.48	0.2
MACE	17.9%	37.4%	3.05	1.43 - 6.42	0.004

CI: confidence interval; MACE: major adverse cardiac events; TSH: thyrotropin.

## Discussion

The major finding of this study supports data previously published, showing that in-hospital MACE of patients with ACS were associated with higher levels of TSH. In addition, we also showed a relationship between TSH and cardiogenic shock and bleeding.

There are many possible pathophysiological explanations for the uncertain relationship between worse prognosis and thyroid hormones in cardiovascular diseases. Numerous studies have focused on the impact of subclinical thyroid dysfunction on the development of cardiovascular disease, especially ACS. However, we do not know if the TSH levels are higher prior to ACS or if they become higher at the moment of ACS.^2,3,7-11^

Triiodothyronine functions through interactions with isoform type α receptors, α1 or α2, and type β receptors, β1, β2 or β3.^2,3,12,13^ Regarding their cardiac distribution, these receptors are located both on atrial cells, as well as on ventricular cells.^2,3,12-14^ By binding to these receptors, thyroid hormones accelerate myosin synthesis and influence sarcoplasmic reticulum activity, movement through the ionic Ca and K channels, response of adrenergic receptors, transmembrane ion gradients, and the levels of ATP and of atrial natriuretic peptide.^2,3,12-14^ The effects of thyroid hormones can be categorized as genomic or non-genomic, and can structurally and functionally influence cardiovascular proteins.^2,3^ Acting on a receptors, triiodothyronine plays a role in the process of increasing myocardial contractility and enhancing myosin production. Acting on β receptors, they influence diastolic processes and left ventricular relaxation. The main mechanism is that of reducing the high levels of cytosolic calcium during systole. On a vascular level, triiodothyronine plays an essential role in the maintenance and renewal of endothelial integrity, in peripheral arterial resistance and in modulating the arterial response to the renin-angiotensin-aldosterone mechanism activation.^2,3,15^ This hormone also controls the macrophage response to the deposition of lipids in the vascular wall.^2,3^ Apart from these direct effects, thyroid hormones play an important role in the development of cardiovascular pathology by other mechanisms, such as influencing the coagulation process by controlling the levels of activated factor VII and the ratio of activated factor VII and anti-activated factor VII antibody.^2,3^

Specifically, hypothyroidism reduces cardiac output, blood volume, chronotropism and inotropism, and increases systemic vascular resistance, diastolic blood pressure, vascular wall thickness and stiffness, and afterload. The increase in peripheral resistance mainly induces left ventricular systolic dysfunction and abnormal relaxation, without modification of heart rate. Changes in arterial wall elasticity are involved in the progression of atherosclerotic processes. Effects on vascular endothelial function alter blood flow, and nitric oxide plays an important role in this process. Hypothyroidism decreases glomerular filtration rate, which influences circulating cholesterol levels and favors the development of type II diabetes complications.^2,3,16,17^ These findings could partially justify the higher occurrence of ACS in this group of patients, and perhaps their worse prognosis. In addition, this mechanism could be associated with the development of cardiogenic shock, well described in our study.

In 2005, Walsh et al.^18^ studied the relationship between thyroid hormone and cardiovascular events in 1,981 healthy individuals in Australia. In a cross-sectional study, they examined the prevalence of coronary heart disease in subjects with and without subclinical thyroid dysfunction. In a longitudinal study, they examined the risk of cardiovascular mortality and coronary heart disease events (fatal and nonfatal). Subjects with subclinical hypothyroidism (n = 119) had a significantly higher prevalence of coronary heart disease than euthyroid subjects (OR = 1.8; 95% CI: 1.0 - 3.1; p = 0.04). In the longitudinal analysis of subjects with subclinical hypothyroidism, 33 coronary heart disease events were observed as compared to 14.7 expected (HR = 1.7; 95% CI: 1.2 - 2.4; p = 0.01).^18^

Another study^1^ in 2005 investigated whether thyroid hormone levels had any predictive value for mortality in patients presenting to the emergency department with AMI. Three groups of patients admitted to the emergency department within the 11-month study period: 95 patients with chest pain and diagnosed AMI; 26 patients with chest pain and no AMI; and 114 controls with no evidence of any major disease. Cardiac enzymes and thyroid hormones were analyzed and compared between groups to examine the effects of historical and demographic factors. Sixteen patients with AMI (16.8%) died within the study period. Troponin and creatine kinase M-type subunit levels were significantly higher among non-survivors as compared with survivors. Survivors in the AMI group had higher levels of triiodothyronine and total thyroxine and lower free thyroxine levels, while non-survivors in the AMI group had higher TSH and lower triiodothyronine, total thyroxine and free thyroxine levels than controls. In logistic regression, TSH levels were not significantly different between survivors and non-survivors (1.08 mIU/L vs. 1.84 mIU/L, p = 0.1). The conclusion was that triiodothyronine and lower free thyroxine appeared to be independent prognostic factors in patients with AMI.^1^ In our study, we showed a trend towards higher levels of troponin and TSH. However, correlation until this moment was not significant. Differences might appear with a larger sample.

On the other hand, in 2014, Him et al.^19^ retrospectively reviewed the relationship between thyroid hormone levels and AMI severity in 40 patients with STEMI, and the extent of transmural involvement was evaluated via contrast-en-hanced cardiac magnetic resonance imaging. The high triiodothyronine group (≥ 68.3 ng/dL) exhibited a significantly greater transmural involvement (late transmural enhancement > 75% after administration of gadolinium contrast agent) than did the low triiodothyronine group (60% vs. 15%, p = 0.003). However, a significant difference was not evident between the high- and low-TSH and free thyroxine groups. When the triiodothyronine cut-off level was set to 68.3 ng/dL using a receiver operating charac teristic curve, the sensitivity was 80% and the specificity was 68% in terms of differ entiating between those with and without transmural involvement.^19^

Friberg et al.^20^ have described a possible rapid down-regulation of thyroid hormones in patients with AMI. Forty-seven consecutive euthyroid patients with AMI were studied prospectively during the first 5 days, and again 6 and 12 weeks after AMI. They observed that the thyroid system was rapidly down-regulated with maximal changes appearing 24 to 36 hours after onset of symptoms. Levels of TSH declined 51% (p < 0.001) between the first 6 hours and the 24 to 36-hour period. The authors also described a strong relationship between inflammation (high levels of C-reactive protein and cytokine interleukin 6) and a greater down-regulation of the thyroid system. Alternatively, MACE were high among patients with the most pronounced TSH depression, indicating that the down-regulation observed after AMI may be maladaptive. Lower TSH levels measured at 5 days significantly correlated with mortality in one year (1.0 mIU/L vs. 1.6 mIU/L, p = 0.04, respectively, between dead and alive patients).^20^ This difference from our results may be because we did not assess TSH levels on the first and fifth days after ACS in our study. Our analysis of only the initial sample at hospital admission was not included in that study by Friberg et al.^20^

Another study has investigated whether changes in plasma thyroid hormone levels were associated with the recovery of cardiac function in patients with AMI. A total of 47 patients with AMI and early reperfusion therapy were included in this study. Cardiac function was assessed by echocardiography; left ventricular ejection fraction and function recovery were evaluated 48 hours and 6 months after AMI. A strong correlation was found between function recovery and total triiodothyronine levels (r = 0.64, p = 10^-6^) 6 months after AMI. Furthermore, multivariate regression analysis revealed that triiodothyronine at 6 months was an independent determinant of ventricular function recovery. TSH levels were not significantly different between the two groups (with and without ventricular function recovery) during the acute phase of myocardial infarction, but at 6 months, TSH levels were significantly higher in the group without recovery as compared with the group with better recovery of ventricular function (2.9 vs. 1.46, p < 0.05).^21^

A study published in 2016 assessed a prospective 3-year cohort with 2430 patients submitted to percutaneous coronary intervention with *versus* without hypothyroidism. The authors related a higher number of MACE (myocardial infarction, stroke, revascularization) in patients with hypothyroidism or TSH > 5.0 mIU/L (HR = 1.28, p = 0.0001).^22^ These data were similar to our findings, but they evaluate long-term prognosis. However, the association with worse prognosis was the same, including the similar value of TSH described.

In summary, different studies have shown a relationship between prognosis and the level of thyroid hormones in ACS. However, the best cut-off, the ideal moment to evaluate TSH levels, and the expected changes after ACS are not known. Combining our results with others from the literature, we postulate that the value of TSH on hospital admission could be helpful and that the prognosis is worse if TSH levels are high at that timepoint. In addition, including the evaluation of other thyroid hormones could be beneficial.

### Limitations

This study showed some limitations, such as the small number of patients evaluated. In addition, we did not measure other thyroid hormones. In addition, this is a retrospective study, and the group with higher TSH levels had worse baseline characteristics, such as higher troponin levels and lower ejection fraction. However, this is an original and novel observation, and other prospective studies will be required.

## Conclusion

In patients with ACS and TSH > 4 mIU/L on hospital admission, worse prognosis was observed, with higher incidences of in-hospital MACE, cardiogenic shock and bleeding events.
